# The 5As team intervention: bridging the knowledge gap in obesity management among primary care practitioners

**DOI:** 10.1186/s13104-015-1685-8

**Published:** 2015-12-22

**Authors:** Ayodele Ogunleye, Adedayo Osunlana, Jodie Asselin, Andrew Cave, Arya Mitra Sharma, Denise Lynn Campbell-Scherer

**Affiliations:** Department of Medicine, Obesity Research and Management University of Alberta, Li Ka Shing Center for Health Research Innovation, Edmonton, AB T6G 2E1 Canada; Department of Family Medicine, University of Alberta, 6-10 University Terrace, Edmonton, AB T6G 2T4 Canada

## Abstract

**Background:**

Despite opportunities for didactic education on obesity management, we still observe low rates of weight management visits in our primary care setting. This paper describes the co-creation by front-line interdisciplinary health care providers and researchers of the 5As Team intervention to improve obesity prevention and management in primary care.

**Methods:**

We describe the theoretical foundations, design, and core elements of the 5AsT intervention, and the process of eliciting practitioners’ self-identified knowledge gaps to inform the curricula for the 5AsT intervention. Themes and topics were identified through facilitated group discussion and a curriculum relevant to this group of practitioners was developed and delivered in a series of 12 workshops.

**Result:**

The research question and approach were co-created with the clinical leadership of the PCN; the PCN committed internal resources and a practice facilitator to the effort. Practice facilitation and learning collaboratives were used in the intervention For the content, front-line providers identified 43 topics, related to 13 themes around obesity assessment and management for which they felt the need for further education and training. These needs included: cultural identity and body image, emotional and mental health, motivation, setting goals, managing expectations, weight-bias, caregiver fatigue, clinic dynamics and team-based care, greater understanding of physiology and the use of a systematic framework for obesity assessment (the “4Ms” of obesity). The content of the 12 intervention sessions were designed based on these themes. There was a strong innovation values fit with the 5AsT intervention, and providers were more comfortable with obesity management following the intervention. The 5AsT intervention, including videos, resources and tools, has been compiled for use by clinical teams and is available online at http://www.obesitynetwork.ca/5As_Team.

**Conclusions:**

Primary care interdisciplinary practitioners perceive important knowledge gaps across a wide range of topics relevant to obesity assessment and management. This description of the intervention provides important information for trial replication. The 5AsT intervention may be a useful aid for primary care teams interested to improve their knowledge of obesity prevention and management.

Clinical Trials.gov (NCT01967797)

## Background

Improving health outcomes for people living with obesity is paramount to healthcare providers and policymakers. This is in part because the annual total costs of obesity in Canada ranges up to $11.08 billion Canadian dollars [1]. Studies suggest that a primary care-based obesity treatment model could be cost-effective over the long term [2]. However, there is a paucity of evidence on the effectiveness of the current obesity management services provided through primary care [3, 4]. The Canadian Obesity Network—Réseau canadien en obésité (CON-RCO) has developed the “5As of obesity management” framework [5], which incorporates the conceptual structure of the best practices in obesity management in a step-wise approach (ask, assess, advise, agree and assist) to facilitate obesity management in primary care [5]. The aim of the 5As Team (5AsT) study is to examine the impact of a team-based intervention on the frequency and quality of obesity management encounters in a primary care setting. [6]

Recently there has been increased awareness on the need for improved reporting of the details of complex innovations being testing in real-world settings in pragmatic study designs [7, 8]. This has led to the international panel from the EQUATOR network creating the TIDieR guide, with the intent to have sufficient detail to permit more nuanced understanding of the context, and content of the intervention [9]. 5AsT is a pragmatic study that seeks to work in real world context, and to create an intervention that works in this setting. Thus, context, and the end-user’s input is crucial in creating the intervention [10]. The focus of this paper is to provide a detailed overview of the 5AsT intervention to support complete reporting and replication.

## Methods

**Fig. 1:**
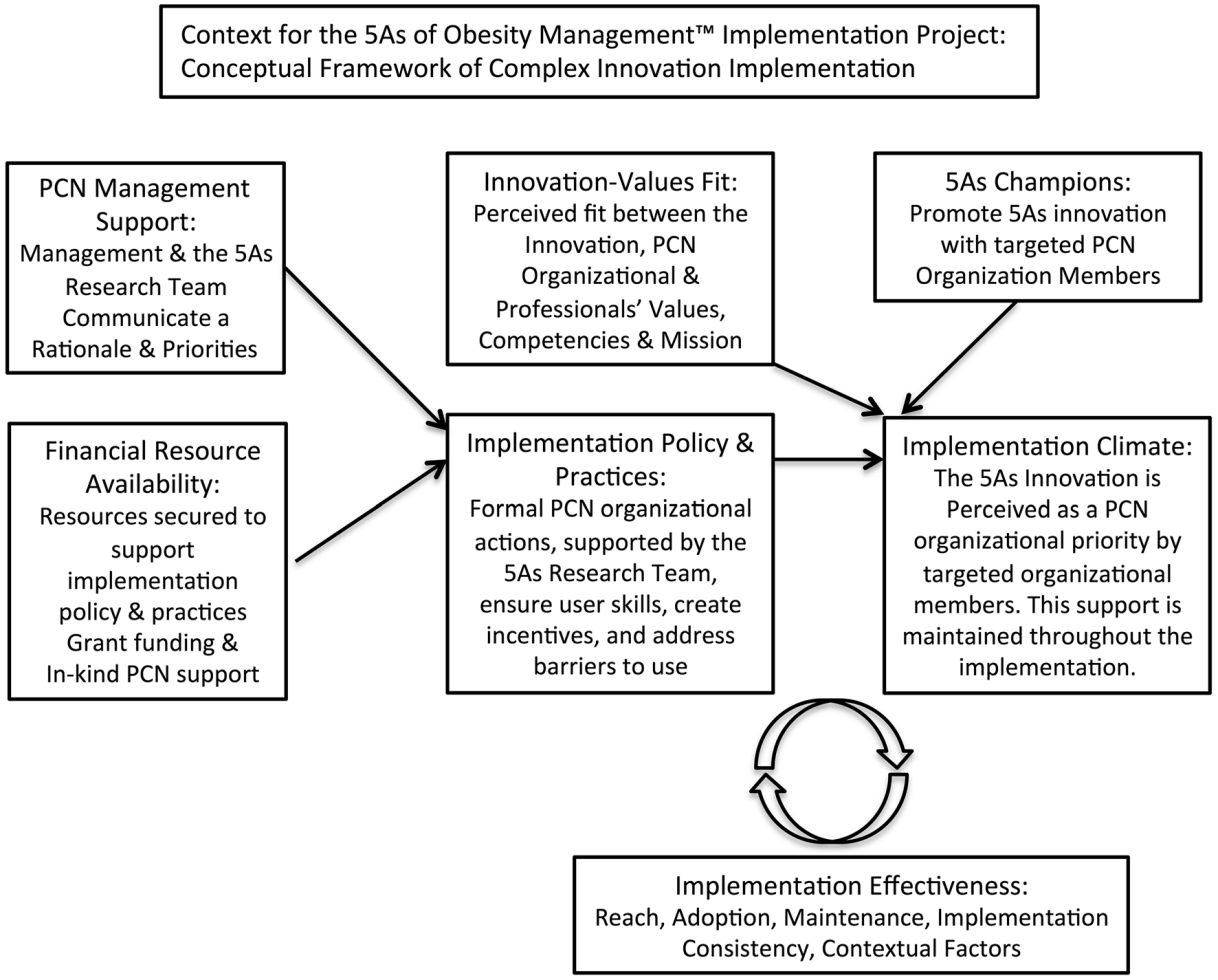
Conceptual framework of complex innovation implementation (adapted from [11])

**Fig. 2:**
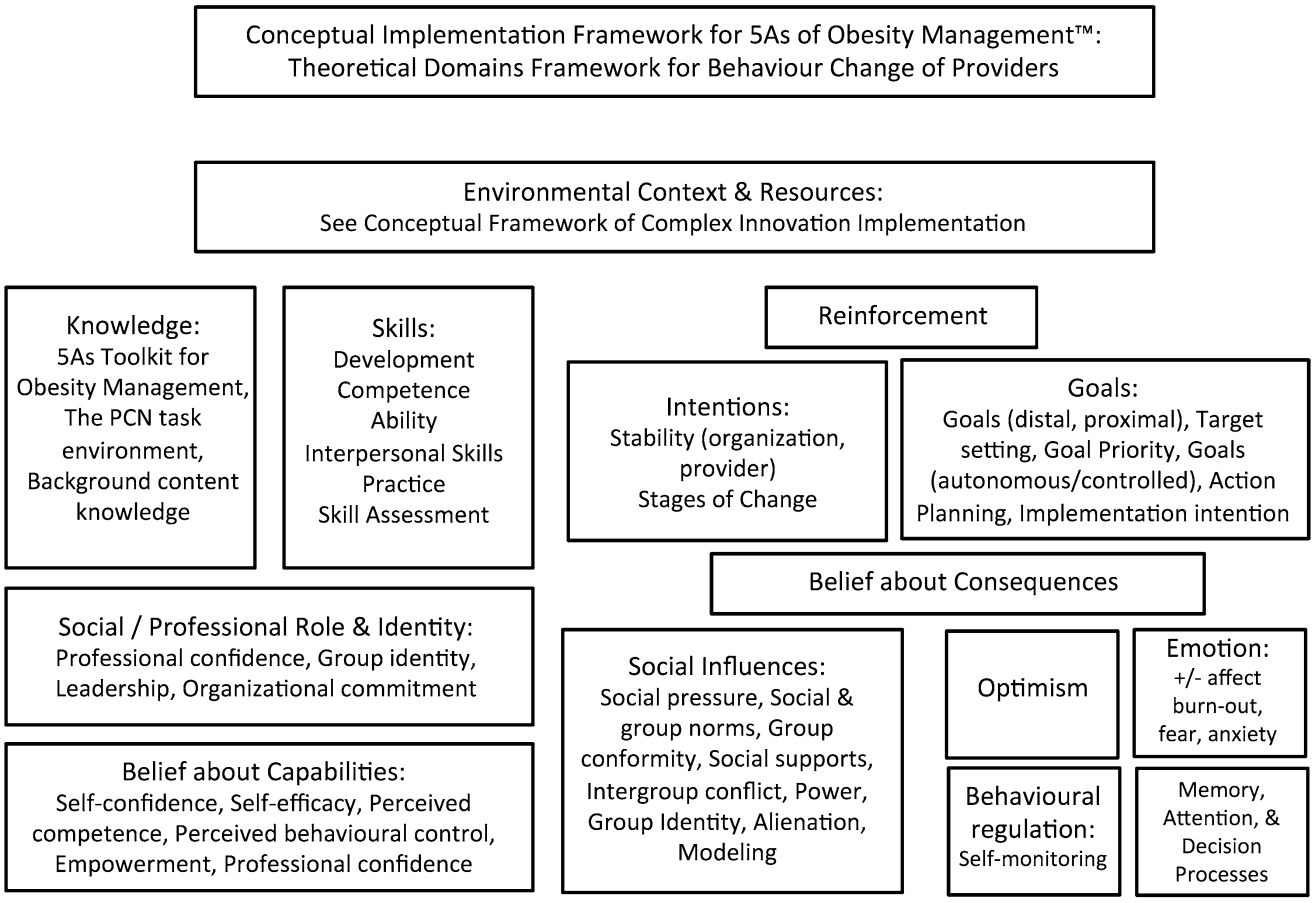
Theoretical domains framework for behaviour change of the provider (adapted from [12])

The intervention was informed by the conceptual framework of Complex Innovation Implementation (CII) [11] and by the Theoretical Domains Framework (TDF) [12], illustrated in Figs. 1 and 2. CII is important because ensuring good alignment with the care organizations’ visions and business plan, increases the likelihood for ongoing stable partnership for the duration of the intervention. The detailed negotiation of the study question, and mode of delivery of the intervention was important as it led to a strong innovations-values fit with the organization and supported the implementation climate. A key insight from CII was the need for a clinical champion, a trusted clinical member of the team, who could act as a liaison between the care organization and the research team. This individual was provided by the partner organization as an in kind contribution, and was crucial for the intervention implementation. The TDF was important as it informed the nature of the intervention as having to include not only knowledge elements, but also deliberate efforts to promote social/professional role identity, and social influences, peer support, practice, and the setting of individual provider goals. This led to the structure of the intervention having a content element, and a learning collaborative element.

This intervention was designed to be tested in a pragmatic randomized control trial with a longitudinal convergent mixed-method design, which has been described in detail in the protocol elsewhere [6]. Briefly, 5AsT is an allocation concealed; pragmatic randomized controlled trial with longitudinal convergent mixed-method evaluation aimed at increasing the number and quality of weight management visits conducted by primary care providers [6]. Of note, there was ongoing monitoring of the intervention delivery, the context and the impact of the intervention using interviews, log books, and field notes [6]. We present here only data pertinent to provider views of the intervention itself.

**Fig. 3:**
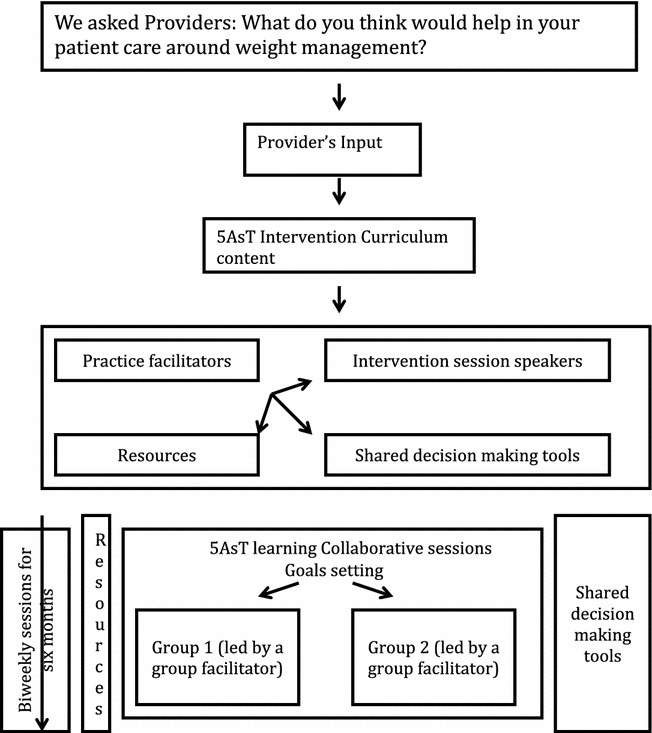
Schematic diagram illustration the 5Ast intervention and its components

Participants in the intervention design were team members from primary care clinics randomized to the 5AsT intervention (Registered Nurses/Nurse Practitioners, Mental health workers, Registered Dieticians), and the researcher team (family physicians, obesity specialist, anthropologist, epidemiologist, public health). In this paper, we describe the derivation of the 5AsT intervention, including the co-creation with the community partners of the research questions, and the process of eliciting practitioners’ self-identified knowledge gaps to inform the curricula for the 5AsT intervention. Themes and topics were identified through facilitated group discussion and a curriculum relevant to this group of practitioners was developed and delivered in a series of 12 workshops. The intervention commenced with a kick-off session October 21, 2013, 12 × 2-h workshop sessions held biweekly for 6 months (November 2013–April 2014); and, an evaluation session post-intervention in May 2014, and 6-months after the end of the intervention (October 2014). See Fig. 3 for a schematic diagram of the 5AsT intervention.

### Study setting

The 5AsT study was conducted in a primary care network (PCN) in Alberta, which employs dedicated multidisciplinary healthcare providers (nurses, nurse practitioners, mental health workers, dieticians, exercise physiologists, respiratory therapists) embedded in 67 family practices with over 170 family physician members serving 192,655 Albertans. This PCN is an extension of the primary care services, which provides a comprehensive family medicine through multi-disciplinary teams that include physicians, nurses, dietitians, social workers, respiratory therapists and exercise specialists. These extended teams are embedded in community family practices and provide support for chronic disease management. As the physicians are fee for service, and the interdisciplinary team members are salaried, it was easier for the team members to participate in this initial intervention. Ongoing work external to this project is ongoing for physicians, evaluating more condensed training formats.

#### Intervention group

The multidisciplinary providers in the clinics randomized to the 5AsT intervention group (n = 29) were consented at each stage of our evaluation (in order to give them the chance to decline participation at any point). All providers were age ≥18 years, one provider was male and all others were female. Six of the providers were registered dieticians, with a seventh new hire joining 1 month into the intervention; seven mental health workers; and 15 registered nurses/nurse practitioners (one withdrew post-randomization). All providers contributed to the design of the intervention.

#### Control group

Providers from the control group were not consented as only de-identified, routinely collected data was used from this group. The control group received standard training in the 5As, as well as other obesity training from the regional health authority, as part of their orientation and development through their employer. They did not receive the 5AsT intervention program; we expected them to continue their standard practice. As they practice in geographically dispersed locations from the intervention team members, contamination was minimized.

## 5AsT intervention

The content of the 5AsT intervention was derived by asking primary care practitioners (n = 29) attending the 3-h kick-off session with an introductory teaching session on the 5As of Obesity Management™, followed by an interactive workshop to determine the content for the intervention. The providers were asked the following question: “What do you think would help in your patient care around weight management?”

Providers identified topics, which were related to themes around obesity assessment, prevention, and management from which they felt the need for further education and training. The 5AsT members then categorized the materials into intervention sessions from the topics [two members (DCS and AAO) initially did the categorization of the topics, which was debated and approved by other team members]. The team, with strong prior relationships with the obesity community, then coordinated with regional experts and resources to find speakers to support each of the 12 intervention sessions.

In the 5AsT intervention sessions an invited speaker presented for about 1 h. They were encouraged to be interactive and to bring useful tools and resources on the topic. The presentation was then followed by a learning collaborative session for an hour, as described below.

As it was expected that not all providers could make each session, eleven sessions were videotaped and posted to You Tube (with presenters’ written consent) immediately after each session. The purpose was to allow for providers to watch the talk if they were not able to make the session. The twelfth sessions was an interactive team communications session for the PCN, so was not videotaped.

Table 1 provides an overview of the intervention content based on the users’ needs assessment, providers/speakers, their expertise and the summaries of the session content. The attendance at each session, by discipline is provided. The intervention materials have been compiled into learning modules and are available at http://www.obesitynetwork.ca/5As_Team.

**Table 1:** Intervention sessions, speaker’s designation and a session summaries of the content

Speakers	Topics	Sessions summaries	Date	Physical attendance~	Breakdown by provider A: RN/NP^a^ B: Dietician C: Mental Health	Evaluation proportion (N = 23) that rated the session 1–3 (n) on 7-point Likert
Bariatric rehabilitation specialist	Weight bias	Explanation of weight bias. Providers should be polite to patients and they should create conducive atmosphere for them in their practice	Nov 7, 2013	23/27	A: 14/14 B: 5/6 C: 4/7	96 % (22)
PCN Dietitian	Emotional eating	Session highlights include: types of hunger drives, reward and stress hunger. Introducing tools that help realign hunger and balance eating Factors that distort hunger cues, inactivity and depression	Nov 21, 2013	20/27	A: 11/14 B: 5/6 C: 4/7	96 % (22)
Registered nurse from Weight Clinic	Clinical assessment of obesity related risk	Speaker mentioned to providers how to assess the readiness to change in patients and the use of checklist for this assessment. And that BMI is a risk assessment index and should not be used for managing the patients or setting goals	Dec 5, 2013	17/27	A: 10/14 B: 4/6 C: 3/7	91 % (21)
Human nutritionist	Pregnancy, post-partum, obesity	Talk was based on promoting healthy weights in pregnancy and strategies to promote healthy eating in pregnant women	Dec 19, 2013	20/28^a^	A: 12/14 B: 6/7^a^ C: 2/7	78 % (18)
Physical activity and exercise specialist	Exercise and weight management	Debunking myth around PA/exercise and the relationship between weight loss, fat mass and fat free mass	Jan 16, 2014	20/28	A: 11/14 B: 6/7 C: 3/7	74 %(17)
Anthropologist	Culture and the body, culture and food —perspectives on obesity	Talk emphasized the important of the cultural perspective of the patient in their dietary intake, weight gain and weight loss	Jan 30, 2014	22/28	A: 11/14 B: 7/7 C:4/7	82 %(18)
Department of Medicine	5As of obesity management	The idea of weight loss plateauing’ was introduced to providers. Strategies on using the 5As of obesity management and critical conversation were highlighted to providers, followed by a providers’ role-play of the 5As card game	Feb 13, 2014	19/28	A: 11/14 B: 6/7 C: 2/7	87 % (20)
Family doctor	Weight gain prevention	The different evidence-based obesity prevention interventions that were available in the literature were shared with providers in this session	Feb 27, 2013	17/28	A: 11/14 B: 5/7 C: 1/7	83 % (19)
Psychologist	How to sustain the change	Providers were told that the goal of obesity management should be about continuous balanced healthy lifestyle and should be focused on sustainable goals. The transtheoretical model (with 5 stages of change) was also highlighted	Mar 13, 2014	20/28	A: 12/14 B: 5/7 C: 3/7	74 % (17)
Psychiatrist	Depression anxiety and obesity	Speaker talked about weight gain following the use of antidepressants. Speaker also encouraged the use of biological, psychological and social evaluation of depression in primary care	Mar 27, 2014	20/28	A: 12/14 B: 5/7 C: 3/7	76 %(16)
Provincial Bariatric Resource Team	Critical conversations	This session was on the importance of common messaging among providers and a focus on tools that can help with key conversations among providers & between providers and patients	April 10, 2014	18/28	A: 11/14 B: 4/7 C: 3/7	60 % (12)
PCN Nurse practitioner and Dietitian	Communication process	The focus was on the ESPCN procedures. The different effective communication strategies, internal process and ideas that improve teamwork among providers were also discussed in this session	April 24, 2014	17/28	A: 9/14 B: 5/7 C: 3/7	50 % (10)

^a^New dietician joined the PCN in an intervention clinic and commenced 19/12/2013

One nurse withdrew from the study post-randomization

2 mental health workers could not physically attend sessions as scheduled when they were off; 1 mental health worker discontinued due to personal leave from work

~All sessions’ content was asynchronously accessible by video for when they could not physically attend. Summaries and resources were emailed to all participants following every session. Hence, providers evaluated sessions whether they participated physically or viewed the video

### Learning collaborations

The advantages of learning collaboration in primary care practice have been highlighted previously [13–15]. Briefly, learning collaboration is a learning process centered on sharing among participants. In other words it is a shared learning process in which participants are responsible for their own learning as well as for one another [16]. It can be a good strategy to leverage resources [17], and also, an important advantage of collaborative learning is to facilitate group learning in order to achieve a particular goal.

The providers were divided into two groups for the learning collaborative, with colleagues working in the same clinic teams grouped together. The learning collaboratives had facilitated discussion of the presentation content of the day, tools and materials shared with them prior to the session, and reflection from their practice experience. At the goal setting element of the session, providers also had the chance to share with the rest of their group the goals they set for themselves and the resources they found useful in their practices.

Some elements of our collaborative learning include: learning about newer research knowledge, practices on weight management and patient goal setting sessions, team-driven small tests of change, collaborative resource sharing among providers, experience sharing teach-backs, and the sessions being led by an experienced facilitator.

### Practice and group facilitators

It is important also to note that we employ the use of practice facilitators and group facilitators in the 5AsT intervention. The use of practice facilitators has been previously described as an effective strategy to improve primary care processes, outcomes, and the delivery of services [18]. Two kinds of practice facilitation were employed in the study: internal (clinical champion) and external practice facilitators. The internal practice facilitator, or clinical champion as informed by the complex innovations framework, was the person designated by the PCN 1 day per week to support the intervention. This was a trusted clinical colleague (dietician) and leader who was able to support the providers in their context, and liaise with the research team to support creating space, climate, and time for the intervention. The external practice facilitators in the 5AsT study acted as a link between providers and evidence or resources that may be used to facilitate weight management encounter with patients as illustrated in Fig. 3. They identified and liaised with speakers, and implemented the planning and execution of the intervention and evaluation session.

Following each session, the external practice facilitators compiled a summary of the materials, and circulated them to the members of the group. In addition, each time a participant identified that it would be useful to have a tool or resource, the external practice facilitators identified one and provided it. Where none existed, they were created with the assistance of a graphic design team, and iteratively reviewed with the participants. This has been described in detail elsewhere, and the tools compiled are available for use [19].

In addition to the practice facilitators, the learning collaboratives had facilitated discussions by the internal practice facilitator, and another trusted internal PCN expert. The group learning collaborative facilitator’s roles was to prompt the conversation among providers and to lead the goal setting sessions. The two group facilitators were rotated on two occasions during the early aspect of the intervention to improve discussion and sharing among providers in the separate groups. This modification was deemed necessary so that the two goal setting groups would experience both group facilitators with their different personal attributes.

### Evaluation of the 5AsT Intervention

The evaluation of the 5AsT intervention was done in three ways: (1) real time monitoring with field notes as described above; (2) individual semi-structured interviews with all participants and (3) questionnaires presented to the participants following the 6-month intervention at the evaluation session.

For the qualitative portion, three researchers took field notes during all sessions. Semi-structured interviews were conducted with all intervention participants (N = 29). The field notes and interviews focused on key aspects of: Theoretical Domains Framework (knowledge, skill, beliefs about capabilities, goals, beliefs about consequences, intentions, emotion, optimism, and role identity) [12], Complex Innovations Implementation (CII) [11], a framework developed to locate and build upon factors that may influence intervention success, and questions pertaining to their views of the intervention, the 5AsT approach and their work environment. We used a thematic analysis approach to determine themes from within the qualitative data [20, 21]. Transcripts were inductively coded line by line according to subject. Data was managed using NVIVO 10 software (QSR International, Burlington, Mass.) Research team members and an independent third party cross-checked all analysis and key findings were shared with participants after the intervention, at which point an opportunity for comment was provided. This paper presents only the results relevant to the evaluation of the intervention.

For the quantitative evaluation we used an intervention specific questionnaire to evaluate the sessions, and a Likert scale to rate each of the intervention sessions and exact data from the providers regarding the intervention. The questionnaire reports a 7-item Likert scale (1-Excellent, 2-very good, 3- good, 4-satisfactory, 5-poor, 6-very poor and 7- unable to comment), at the evaluation session on May 8, 2014. Quantitative data was managed in Microsoft Excel and analyzed in SPSS software.

This study is approved by the University of Alberta ethics committee and was registered at Trials.gov (NCT01967797). It is funded by an Alberta Innovates Health Solutions grant, with significant in kind support from the Edmonton Southside Primary Care Network.

## Results

Providers identified 43 topics that they thought would be helpful in their patient conversations about weight management at the kick-off session (“Appendix 1”). These topics were grouped into 13 themes, which facilitated the choice of 5AsT intervention speakers and the content of the 12 sessions Table 1). The topics for the 12 sessions (“Appendix 2”) are related obesity assessment and weight management in which practitioners felt the need for further education and training. These included issues related to cultural identity and body image, emotional and mental health, motivation, setting goals, managing expectations, weight-bias, caregiver fatigue, clinic dynamics and team-based care. Participants also identified a need for greater understanding of physiology and the use of a systematic framework for obesity assessment (the “4Ms” of obesity).

The attendance sheet was used as a proxy to measure adherence of the participants to the intervention. Detailed attendance by session is reported in Table 1. Fifteen providers attended ≥10 sessions of the intervention, including five who attended all sessions. Nine providers attended 5–9 sessions. Five providers attended fewer than 5 sessions including: one who withdrew from the study at the beginning (no data), two mental health workers did not attend the any sessions, and two who only attended a few sessions. All providers contributed to the interviews.

At the final evaluation session on May 8, 2014, 21 providers (9 = RN, 3 = NP, 2 = MHP, 7 = RD) were present on the day and two addition providers filled the questionnaire and returned it on a subsequent date.

On the 7-item Likert scale, 83 % of respondents rate the intervention as either very good or excellent, with the remaining 17 % rating it as good. Overall, 86 % of the providers responding also said they were either strongly comfortable or somewhat comfortable with the 5As of Obesity Management™ [5] following the 5AsT intervention, and 91 % reported they felt more comfortable discussing weight issues with their patients as a result of the intervention. Of the 23 respondents, 21 reported they would recommend the intervention to others, and 2 respondents felt they were not able to comment.

In terms of the structure of the intervention, overall, 18 of the 23 respondents (82 %) felt that biweekly (once in 2 weeks) learning collaboration format was suitable for them. Table 1 provides the proportion of the 23 respondents that scored each session excellent, very good, or good (1–3) on the Likert scale.

In terms of the learning collaborative groups, 73 % (16) of the respondents rated them as excellent/very good/good. Of the respondents, 64 % felt the goal setting in the learning collaborative sessions was helpful, with 39 % reporting that they often/always met their goals.

The Youtube videos were used by 64 % of respondents, and among those who viewed them 87 % rated the videos as very good or good. The main challenge was the sound quality of the videos.

Overall, the intervention was very well received, Interview and field note data reveal strong intervention values fit and self-reported behavior change. Table 2 provides some representative quotes of positive views of the intervention, while Table 3 provides some examples of challenges from provider views of the intervention. The overall results are summarized below.

**Table 2:** Examples of representative positive provider views on the intervention

*“I like the way that is set up, I like the tools, I like, I do like the, actually it’s all been good. I mean I’ve really, I’ve enjoyed the presentations, you know I’ve, I’ve gotten, I’ve taken something back from each of them, there’s no question and I think it’s unrealistic to expect that you can put out a kind of an itemized sort of what do you call it? Like a flow for sort of how you’re going to, it’s not going to work the same in every clinic, not going to work the same right so I think that’s unrealistic expectation. I think what you’re, how the way you’re approaching it is much better, here’s the concept, here’s, you know here’s a variety of tools you know but the general idea is this, you know take it and mold it to work in your clinic or mold it; yeah I think that’s the best approach because it has to be flexible, it has to be”* A4, nurse	
*“I thought it was very good. I especially enjoyed today. I think it gives us new ways to look at things and I think we need each other’s ideas because lots of times there’s just one little thing that somebody else does that you never thought of and if we, if we work in isolation, you know if we never have meetings then and we always do the same thing with patients, we don’t get any new ideas and I think that’s important in learning, you know trying different things. Maybe it won’t work but at least you’ve tried or, or it gives you another idea… Yeah I like that a lot. I like some sort of formal presentation. I, I, I need, I think we need a bit of structure and so the first part is structured, the next part is not and I, I kind of like, actually like the idea of smaller groups. I think people are not as willing to, to open up in a large group and I’m sure we’ll find that, you know myself included”* A5, nurse	
*“Yeah, really good and I’ve been at all of them and I found they all, were all really good. I find some of it repetitive, like some of it is I find might be a little bit more like it’s kind of the same things over and over again but it’s good, it’s good. It gets you thinking and it, and I think it’s good that it’s ongoing ‘cause otherwise you take a course and you’re good for a week and then you kind of go yeah I kind of forget about that you know more as time goes on whereas this is kind of reinforcing it, instead it’s becoming more a part of your practice if you weren’t already doing that to start off”* A9, nurse	
*“Yeah, yeah, they’re really good. The only thing I’d change maybe is it’s tough for Thursday mornings, sometimes I’m busy at the clinic and it’s tough to get that time off ‘cause I’m used a lot on the spot here so sometimes I’ll have a bunch of appointments and sometimes I won’t but I’m always just kind of pulled onto the floor and so it’s tough to get that time away so I don’t know what else we could really do but especially for people that have clinics way all over… I, I like the breakout afterwards and then we can kind of discuss it as a smaller group ‘cause then it makes it a little bit easier for people to talk I think as well to facilitate that. Yeah ‘cause when bigger groups, it’s harder to… I like that you guys ask us what our needs are and, and, and that kind of helps bring in what, what’s relevant to us”* A11, nurse	
*“I find the sessions are really helpful. I really like the speakers, I like having the variety of the types of topics that they’re talking about and that’s really important and having the group discussion from a variety of different health professionals is really interesting because it’s easy to just get your dietician perspective so it’s nice to get it from a nursing or from a mental health perspective or by anything like that so… I think it’s, it’s interesting because it gives you enough time to sort of reflect on what you’ve been talking about that for that session. I’m not sure, I, I really just find that the presentations are really nice because that I find that we just don’t get enough of that type of thing so and especially for someone like me who’s relatively new in my practice, I find that it is really helpful to kind of get that type of educational piece”* B1, dietician	
*“The ones, like I said I missed the two but the ones that I went to I found are really useful. I think it’s an area that being mental health it’s not something you always get educated in in school so the things that I’ve learnt so far I think have been really useful … I’m excited about some of it so yeah it’s been interesting. I missed the pregnancy one and that’s the one I think I have to, there’s a link on …we should watch the YouTube video. It’s really interesting stuff so so far everything I’ve learned I think has been applicable”* C6, mental health worker

**Table 3:** Examples of challenges from provider views of the intervention

*“I think it’s good. It’s really good. I just find it’s a little bit long and it pulls us away from our clinics quite a bit and I know that’s a contentious issue with Dr. X that I’m not there as often… and that I’m part*-*time so I have to find a way to give that time, I find maybe if it was condensed maybe a little more, it might be a little more applicable. I, I don’t know”* A2, nurse	
*“Well I’m really excited about it. I mean I, I live the experience of being overweight myself and what a struggle it is, you know but I get the sense that it’s allowing us to explore and really putting out there, it’s giving us a framework to work within even if we’re dealing with our own things and that allows us to, to be better when we’re looking at our clients This week has, has gone so quick and yeah, I mean I guess my only, the only regret is that time away from the clinic but knowing that in the end of it all or though the process of it all, as I acquire more, more knowledge about myself and the program, I will be able to bring that value back to the clinic”* A3 nurse	
*“I’ve been really enjoying them. Some things I find are really new. Other things are refreshers but refreshers are always good. Just collaborating at the end, having an open discussion, getting perspectives from different health care professionals is always good too and like even for today, we identified gaps in terms of the classes that we were offering for nutrition so it brought to light something like change right that can happen so it’s good. I’ve, I’ve really enjoyed it… I mean it’s definitely time consuming and normally that’s not a big issue is just because it’s taking time away from clinic so to me it’s not a problem. The only problem that had come up was because it’s always the same time slot, like the two Thursdays every month, it, it affects the same clinic each and every time so this particular clinic is actually _______ and I’m only there two days a month so this takes out half a day twice a month so then I got a call a few weeks ago saying a patient really wanted to see me, it was kind of like an urgent issue but I wasn’t available until like February so because it affected the same clinic each and every time, it presented an issue but normally I wouldn’t have said that it would have been a problem at all… I really like that you know we kind of get like an education session and then a chance to kind of brainstorm, discuss afterwards”* B4, dietician	
*“Definitely an interest. I mean some of the speakers that we’ve had have been really great and I mean I am learning things from that perspective. How much is applicable, again people aren’t coming to see me specifically for weight management…I wouldn’t say I’ve had a hard time because the clinics are very accommodating and I’ve just booked it out of my schedule, however that, for me that is probably on a Thursday every two weeks, that’s probably anywhere from five to six patients that I could have been seeing right ‘cause, ‘cause I see on average about 10 or 12 a day so it, it, just in, in that respect. Nobody has, nobody has said anything or complained about it but I, I feel it”* C3, mental health worker	
*[Regarding the learning collaborative prior to the re*-*organization]* *“I think the [group] facilitator should rotate or I don’t think you’re going, I think the group altogether is too big so I think they should try to rematch the groups a bit because there’s certain, like the group I’m in is a very quiet group … and you know I’m not going to, I could pipe up a lot but I’m not going to do that right so whereas the other group has a lot of really talkative verbal people so I think they need to either remix it or maybe alternate facilitators. That might be an option”* A10, nurse	
*[Initial skepticism of the front*-*line providers, highlights importance of monitoring internally and provider*-*centred intervention]* *“I think it’s great. I think I’ve said that enough. I initially thought what am I, what have I been pushed into, what are we going to do here and I think a lot of us had that feeling actually because we did discuss it, we’re thinking what are put up, what are we going to do but as it is going on, I think it’s great…* *[Regarding the learning collaborative prior to the reorganization]* *“No I think what I take away from these meetings is a lot. Apart from the actual when we divide into groups [learning collaboratives], I don’t find that beneficial at all except for the last one we did was better but I don’t know, I was having a very difficult time and even realizing while we were sitting in that group and that’s why I had asked can we sit together as one big group ‘cause it seems like when we, every time we’d come back in the room, they [the other learning collaborative group] had this amazing conversation going on about what’s, what they’re supposed to be doing and it felt like we weren’t getting that and I thought then why are we here if we can’t get the full picture and the full education”* A15, nurse	
*“No, so far I’m really enjoying it. There’s been a, like maybe one of the talks where they used terms like what was it? I don’t think I’d want to put it wrong but almost like taking that parenting role with the patient, that really does not fit well with my approach and sort of is against the grain. I, I mean I understand what was meant but I think putting it in those terms perhaps isn’t the best way of explaining it. You, you definitely don’t want to take that approach on patients. I wouldn’t go over well at all or at least not from my experience. Other than that, that’s kind of the only thing that I went “oh” about. I really enjoyed it a lot more than I thought I would enjoy it and I think for the most part it has been, even in a lot of the mental health tools and things that I have, these are much more looking at that whole biopsychosocial perspective for patients, not focusing on calories, not focusing on numbers, that kind of thing and even the tools that I have still sort of reference that so”* C8, mental health worker	

Positive themes that stood out included: variety, it was collaborative, multidisciplinary, long-term and sustainable in that it leveraged the internal practice facilitator as a change agent with the task of ongoing training of new staff in the organization. Comments included appreciating the insights of multidisciplinary teams, hearing their “clinical peers”, sharing ideas, hearing from diverse speakers, and collaboratively discussing issues. One nurse suggested the intervention provides options of where to start the conversation and has changed how in general she thinks about weight management. The format was generally considered positively; providers stated that the recurrent sessions helped the information sink in and gave them time to adapt it to their practice. Participants felt this lead to increase in confidence and comfort with the material. A provider also, suggested that the format of the sessions allowed for self-reflection, with another stating that the structure of the sessions allowed new information to become part of the practice.

Some providers, however, felt that either the sessions were too long, or that it was difficult to get the time away from their clinical practice. The perceived usefulness of the learning collaborative was mixed, many participants feeling that it was both useful to have space to share their clinical experience with peers while also stating that at times the conversation was difficult. However, the structure did lead to increased collaboration between multidisciplinary team members. Active monitoring of the field notes of the intervention meant that the research team was aware of the concerns for the imbalance between the two learning collaborative groups, with one group with more quiet individuals. This was then purposefully reviewed with the group and solutions were obtained from the participants. This led to a rebalancing of the teams between the groups to have more balance, as well as periodic rotation of facilitators.

## Discussion

Through the 5AsT study we were able to identify obesity management related topics and learning that may help providers change behavior, improve their practices and refine obesity encounter for patients. Here we highlight the 5AsT method and intervention content. The intervention sessions, video links and the tools co-created with providers are available on the web (http://www.obesitynetwork.ca/5As_Team). The purpose of these modules is to create a living repository of tools and resources to support primary care teams in the community who would like to improve obesity management in their context. From a research perspective, they serve as a record of the content of the intervention, supporting transparency of reporting [7–9]. Our overarching aim is not only to improve the quantity of obesity management in primary care setting, but also to improve its encounter quality. Through the kick-off of the 5AsT intervention, we identified primary care providers’ barriers and knowledge gaps to weight management in their practices. We envisage that a participatory provider engagement, such as 5AsT intervention, may increase the frequency, quality of weight management encounters in family practices and the quality of life of the patients.

Interventions aimed at changing provider behavior in the real world are best informed by the active engagement of the end-user to ensure applicability and context-appropriateness [10], as was amply observed in this study. The engagement with the end users resulted in many pragmatic solutions to challenges in implementation, which proved crucial. Both the complex innovation framework [11] and the theoretical domains framework informed this intervention [12], with core elements such as practice facilitation (internal [11], and external [18]), proving crucial, and learning collaboratives [13–15] proving more mixed. Overall, the intervention proved positive for the majority of the participants, resulting in self-reported practice change. Challenges frequently revolved around scheduling and time constraints, which were partly mitigated by providing an asynchronous video option for catching up on missed material.

Previous studies suggest that providers experience barriers in obesity management [22, 23] and lack adequate weight management knowledge [24, 25]. We also know the frequency of obesity management in the PCN is low (Unpublished data from routine continuous administrative monitoring), leading to the premise that if we reduce the knowledge gaps in providers we may improve the quality and frequency of obesity management visits by patients and also improve weight management consultations.

Most behavioral weight loss interventions have failed to demonstrate long-term effectiveness and sustainability of weight management. It may therefore be important to encourage more emphasis on other non-weight related outcomes of obesity management intervention as this unrealistic concentration on weight loss by providers, was a key learning point in the course of our intervention. Providers may need to look beyond the anthropometric changes following an intervention and mindful on the quality of life of the patient as well [26]. A key finding was the providers’ choice of topics around caregiver fatigue, relapse prevention, emotional eating, and mental health concerns; daily challenges in their practice.

There are several limitations to this study. The 5AsT intervention can be generalized to other similar populations to a certain extent. Similar to the finding of other studies [23, 27, 28], the knowledge gaps highlighted by the providers involved in this study are common. However, one challenge in our context was it was not possible to include fee for service busy family physicians in the intensive intervention. We were able to have two family physicians participate on the research team. Our future research will focus on interventions on family physicians, and on other aspects of provider’s consultations that may indirectly affect weight management. A primary care system, with a multidisciplinary team, similar to that of 5AsT study is likely to share the same issues as our practitioners have highlighted. However, given the diversity of contexts in which primary care is practiced, future work will need to consider how the intervention may need to be modified for different settings. A rich description of the intervention is a necessary first starting point in synthesizing what works in diverse settings.

## Conclusion

Primary care practitioners perceive important knowledge gaps across a wide range of topics relevant to obesity assessment and management. The 5AsT intervention was designed to respond to the identified needs of front line providers in terms of content, and the structure promoted interaction and collaboration, emphasizing practice opportunities and innovation.

Further work should focus on how these knowledge gaps can be addressed and whether increased knowledge and competencies in these areas will translate into better health outcomes for overweight/obese clients. Furthermore, 5AsT intervention’s goal is improved weight management by improving provider’s knowledge and patients experience. Ultimately, the 5AsT intervention is a promising primary care-based approach co-created with end users to achieve better management of obesity. The 5AsT web resources can support community primary care teams in practice-based learning to improve obesity management.

## Appendix 1: Identified topics from the 5AsT intervention kick-off session

**Table:**

Medication, side effect i.e. weight gain excuses	
Conversations with physicians	
How to get patients to buy in/stay engaged (even after programs)	
How to deflect from a weight goal to a health outcome goal	
Cultural aspect/diet/body image	
Mental health and obesity	
Handling patients emotional issues	
Clinic processes and team based care	
Patients follow-up	
Cultural and identity (in relation to food and body)	
Weight bias	
Caregiver fatigue	
Body image	
Emotional eating	
Behavior change for patient	
Eating disorders	
Sharing stories of success (provider and patient experiences)	
Behavior change smart goals	
Motivational interviewing	
Resources for patient education/where to send	
Resources around physiology (obesity)	
Messaging regarding being proactive	
Establishing collaborating framework/rules	
How to deal with emotional stress/issues	
Caregiver fatigue	
Success stories	
Setting goals on behaviors	
Motivation interviewing	
Recognizing mental health issues	
Body image	
How to use the 4 ‘M’ frame work	
Guideline of questions-how to change practices	
How to keep patients sustaining goals over the long terms	
Appropriate referrals	
How to work with emotional eating	
How to involve families/support/saboteurs	
Patient education on weight loss expectations	
Operationalizing the assessment piece of the 5A to avoid patients and provider fatigue, provider tools, assessment brought up too many issues	
Child and adolescent-an approach to parenting/pregnancy	
Group dynamics	
Prevention/predicting weight gain	
Patients types: active gainer/stable/post weight loss/yoyo: broad group assessment that this needs different approach	

## Appendix 2: The 13 themes derived from the topics Identified by providers in the study

- 5As of obesity management
- Pregnancy and post-partum obesity prevention and management
- Emotional eating
- Clinical assessment of obesity related risk
- Weight bias
- Cultural identity and body image
- Goal setting and managing expectations
- Caregiver’s fatigue
- Clinical dynamics and team-based care
- Critical conversations
- Weight gain prevention
- Depression, anxiety and obesity
- How to sustain the change.

## Abbreviations

5As: ask, assess, advice, agree, and assist
5AsT: 5As team
PCN: primary care network
Equation 5D: European quality of life- 5 dimensions
BMI: body mass index
FTE: full-time equivalent
4Ms of obesity: mental, mechanical, metabolic, and monetary
CON-RCO: Canadian Obesity Network—Réseau canadien en obésité
