# Erratum to: The 5As team intervention: bridging the knowledge gap in obesity management among primary care practitioners

**DOI:** 10.1186/s13104-016-1930-9

**Published:** 2016-03-14

**Authors:** Ayodele A. Ogunleye, Adedayo Osunlana, Jodie Asselin, Andrew Cave, Arya Mitra Sharma, Denise Lynn Campbell-Scherer

**Affiliations:** Department of Medicine, Obesity Research and Management, Li Ka Shing Center for Health Research Innovation, University of Alberta, Edmonton, AB T6G 2E1 Canada; Department of Family Medicine, University of Alberta, 6-10 University Terrace, Edmonton, AB T6G 2T4 Canada

## Erratum to: BMC Res Notes (2015) 8:810 DOI 10.1186/s13104-015-1685-8

Unfortunately, the original version of this article [1] contained an error. The author’s name, ‘Ayodele A. Ogunleye’ did not include the author’s middle initial. This has been corrected in the original article and can also be found correctly in the author list above.
